# The Role of Emotion Dysregulation, Impulsiveness and Rumination in Psychopathology: Evidence from an Italian Sample of Adolescents and Young Adults

**DOI:** 10.3390/brainsci16060620

**Published:** 2026-06-10

**Authors:** Sofia Francesca Aprile, Chiara Avanzato, Francesca Alù, Carmen Concerto, Pierfelice Cutrufelli, Ludovico Mineo, Gabriele Privitera, Antonino Petralia, Alessandro Rodolico, Filippo Caraci, Maria Salvina Signorelli

**Affiliations:** 1 Section of Psychology, Department of Educational Sciences, University of Catania, Via Teatro Greco 84, 95124 Catania, Italy; sofia.aprile@phd.unict.it; 2 Department of Psychiatry and Psychotherapy, Klinikum Rechts der Isar, TUM School of Medicine and Health, Technical University of Munich, Ismaninger Straße 22, 81675 Munich, Germany; alessandro.rodolico@tum.de; 3 Psychiatry Unit, Department of Clinical and Experimental Medicine, University of Catania, Via Santa Sofia 78, 95123 Catania, Italy; chi4493b@gmail.com (C.A.); francescaalu.492@gmail.com (F.A.); carmen.concerto@unict.it (C.C.); pierfelicecutrufelli@yahoo.it (P.C.); ludwig.mineo@gmail.com (L.M.); gabrieleprivitera@libero.it (G.P.); petralia@unict.it (A.P.); 4 German Center for Mental Health, Partner site Munich-Augsburg, 80336 Munich, Germany; 5 Department of Drug and Health Sciences, University of Catania, Viale Andrea Doria 6, 95123 Catania, Italy; filippo.caraci@unict.it; 6 Oasi Research Institute-IRCCS, Via Conte Ruggero 73, 94018 Troina, Italy

**Keywords:** emotion dysregulation, impulsiveness, rumination, psychopathology, adolescence, youth

## Abstract

**Highlights:**

**What are the main findings?**

- In first-time psychiatric outpatients aged 16–25 years, rumination, emotion dysregulation, and impulsiveness were associated with psychopathological symptom dimensions.
- Rumination showed the broadest pattern of associations, whereas emotion dysregulation and impulsiveness showed more domain-specific associations.

**What are the implications of the main findings?**

- Early clinical assessment may benefit from considering rumination alongside emotion-regulation and inhibitory-control difficulties.
- These findings may inform hypotheses for future transdiagnostic interventions targeting self-regulatory processes in youth mental health care.

**Abstract:**

Background: Adolescence and emerging adulthood are periods of heightened risk for the onset of psychopathology. Emotion dysregulation (ED), impulsiveness, and rumination are transdiagnostic processes implicated across symptom domains, but their relative contributions in clinical youth samples remain unclear. This study examined these three self-regulatory processes simultaneously in first-time help-seeking adolescents and young adults, addressing their differential associations with psychopathological symptom dimensions at an early stage of clinical contact. Methods: We conducted a retrospective observational study with a cross-sectional design in outpatients aged 16–25 years presenting for a first-time psychiatric outpatient consultation at a university hospital in Catania, Italy. Participants completed self-report measures of rumination (RRS), impulsiveness (BIS-11), ED (DERS), and psychopathological symptoms (SCL-90-R). Associations were examined using Spearman’s and partial correlations. Multivariate multiple regression, followed by outcome-specific multiple linear regression models, assessed the relative associations of rumination, ED, and impulsiveness across symptom dimensions. Results: Rumination showed the broadest pattern of unique associations, with FDR-significant associations with eight SCL-90-R domains; the largest coefficient was observed for depression (B = 0.053, 95% CI [0.042, 0.064]). ED was associated with obsessive–compulsive symptoms, depression, anxiety, anger–hostility, and phobic anxiety. Impulsiveness showed more limited associations, remaining significant for anger–hostility and psychoticism. Conclusions: Findings are consistent with a transdiagnostic framework in which rumination and ED relate to multiple symptom dimensions, whereas impulsiveness shows more domain-specific associations. Because the study was cross-sectional and based on self-report measures, these associations should be tested in larger longitudinal and multimethod studies.

## 1. Introduction

Adolescence and early adulthood constitute sensitive developmental periods during which psychopathological symptoms frequently first emerge or escalate, thereby influencing the course of mental health across the lifespan [1]. Large-scale evidence indicates that the onset of mental disorders is concentrated early in life, with about one-third beginning by age 14, nearly half by age 18, and roughly two-thirds by age 25, underscoring adolescence and emerging adulthood as a key window for prevention and early intervention [2]. At the population level, mental disorders are also a major contributor to disability in young people: the World Health Organization estimates that around 1 in 7 adolescents experiences a mental disorder, and these conditions account for a substantial share of disease burden in this age group [3]. Nevertheless, many psychiatric disorders remain undiagnosed and inadequately treated, with unmet clinical needs often persisting into adulthood [4]. Within this framework, emotion dysregulation (ED), broadly conceptualized as maladaptive difficulties in modulating emotional experiences and their behavioral and physiological expressions, has been consistently associated with functional impairment and heightened risk across a range of youth psychopathology [5]. Consistent with this, ED and emotion-driven behavioral outbursts are among the most common reasons for referral to child and adolescent mental health services, underscoring the construct’s clinical relevance in routine care [6]. Alongside ED, impulsiveness and rumination represent two additional transdiagnostic processes that are highly relevant during adolescence and emerging adulthood. Impulsiveness, often conceptualized as a tendency toward rapid, poorly controlled responses and reduced inhibitory control, has been linked to a broad spectrum of psychopathological onsets, particularly externalizing problems (e.g., conduct problems, substance use) but also internalizing symptoms (such as depression and anxiety), consistent with the view that impulsivity reflects a core self-regulatory vulnerability [7,8]. In parallel, rumination, typically defined as repetitive, passive, negatively balanced, self-focused thinking, represents a robust risk factor for the onset and maintenance of depressive and anxiety symptoms and has been widely conceptualized as a transdiagnostic mechanism contributing to comorbidity [9,10]. These processes are likely mutually reinforcing [10]. Difficulties in emotion regulation may heighten and prolong negative affect and stress reactivity, which can bias cognitive processing toward perseverative, stress-reactive rumination. Concurrently, sustained emotional arousal may tax executive control and increase the propensity for emotion-driven impulsive behaviors, thereby linking ED to both internalizing and externalizing manifestations of distress [11,12]. Although ED, impulsiveness, and rumination have each been examined as transdiagnostic processes, fewer studies have modeled them simultaneously in adolescents and young adults presenting for first-time psychiatric consultation. These constructs are theoretically related but not interchangeable: ED refers to difficulties modulating emotional responses; impulsiveness reflects a tendency toward rapid, insufficiently controlled responses; and rumination denotes repetitive, passive, negative self-focused thinking. Examining them together allows the relative contribution of shared self-regulatory vulnerability and more specific cognitive or behavioral processes to be compared across symptom domains. Therefore, this study aimed to examine the unique and relative associations of emotion dysregulation, impulsiveness, and rumination with psychopathological symptom dimensions in adolescents and young adults aged 16–25 years assessed during an initial psychiatric outpatient consultation.

## 2. Materials and Methods

### 2.1. Participants

We conducted a retrospective observational study with a cross-sectional design between November 2022 and March 2023. Available records were screened for adolescents and young adults aged 16–25 years who presented for the first time to the psychiatric outpatient services of the “G. Rodolico” University Hospital (Catania, Italy). Records were included if they met the predefined inclusion criteria and contained complete psychometric assessment data. Participants were included on the basis of first-time psychiatric outpatient access, while ED, impulsivity, rumination, and psychopathological symptoms were assessed dimensionally using standardized psychometric instruments administered by trained clinicians as part of the psychiatric consultation.

- Inclusion criteria were (a) age 16–25 years; (b) outpatient status; (c) first-ever access to the “G. Rodolico” University Hospital psychiatric services during the study period; and (d) no previous psychopharmacological treatment.
- Exclusion criteria were (a) neurological comorbidities; (b) intellectual disability; and (c) substance abuse. Individuals meeting any exclusion criteria were not enrolled to reduce potential confounding and to better characterize psychiatric diagnoses independent of these conditions.

Because the study was based on retrospective review of available clinical records, the total number of first-time consultations during the study period, as well as the number of individuals excluded before record inclusion or who did not complete the assessment, was not systematically available. All procedures were conducted in accordance with the ethical standards of the relevant national and institutional committees on human experimentation and with the Declaration of Helsinki (1975, revised 2013). The study received ethical approval from the Institutional Review Board of the University of Catania (protocol IERB-EdUnict-20240603/03). All participants provided written informed consent for the use of their assessment data for research purposes. Data were collected in private rooms and stored in secure, password-protected databases to ensure confidentiality. The study is reported in accordance with the strengthening the reporting of observational studies in epidemiology (STROBE) guidelines for cross-sectional studies (see Supplementary Data, Table S1).

### 2.2. Psychometric Assessment

Surveys were administered digitally and completed in <30 min. Participation was voluntary and uncompensated. Responses were collected anonymously by assigning each participant a unique identification code to avoid duplicate entries. De-identified data were securely transmitted and stored in a password-protected electronic database to preserve confidentiality and data integrity. The survey comprised three sections: (1) study information, informed consent, and researcher contact details; (2) socio-demographic characteristics (age, sex, education, work status); and (3) the standardized instruments described below.

#### 2.2.1. Ruminative Response Scale (RRS)

The Ruminative Response Scale (RRS) is a 22-item self-report measure of ruminative thinking, comprising three subscales (Depression, Brooding, and Reflection) [13]. Items are rated on a 4-point Likert scale from 1 (“never”) to 4 (“always”), yielding a total score range of 22–88; higher scores indicate greater rumination. The Italian version was used for this study [14].

#### 2.2.2. Barratt Impulsiveness Scale (BIS-11)

The Barratt Impulsiveness Scale (BIS-11) is a 30-item self-report questionnaire assessing trait impulsivity [15]. Items are rated on a 4-point Likert scale from 1 (“rarely/never”) to 4 (“almost always”), yielding a total score range of 30–120; higher scores indicate greater impulsivity. We used the Italian version of this scale [16].

#### 2.2.3. Difficulties in Emotion Regulation Scale (DERS)

The Difficulties in Emotion Regulation Scale (DERS) is a 36-item self-report measure of emotion-regulation difficulties [17]. Items are rated on a 5-point scale from 0 (“almost never”) to 4 (“almost always”), with higher scores indicating greater difficulties in emotion regulation. The scale yields a total score and six subscale scores: Emotional Awareness, Emotional Clarity, Emotional Acceptance, Goal-Directed Behavior, Impulse Control, and Emotion Regulation Strategies. The Italian version was used in this study [18].

#### 2.2.4. Symptom Checklist-90-Revised (SCL-90-R)

Psychiatric symptom severity was assessed using the Italian version of the Symptom Checklist-90-Revised (SCL-90-R), a 90-item self-report questionnaire rated on a 5-point scale from 0 (“not at all”) to 4 (“extremely/often”) [18,19]. The SCL-90-R provides scores across nine symptom dimensions, i.e., Somatization, Obsessive–Compulsive symptoms, Interpersonal Sensitivity, Depression, Anxiety, Hostility, Phobic Anxiety, Paranoid Ideation, and Psychoticism, plus seven additional items which are not included in the other subscales. Scores are calculated as the mean of items within each domain (range: 0–4). An overall index of psychological distress, the Global Severity Index (GSI), is computed as the mean score of all items.

### 2.3. Data Analysis

#### 2.3.1. Power Analysis and Preliminary Checks

An a priori power analysis was conducted in G*Power 3.1.9.4 for a two-tailed bivariate correlation, assuming an expected effect size of r = 0.30, α = 0.05, and power = 0.80. This analysis indicated that a minimum sample size of 84 participants was required; therefore, the final sample size of 102 participants was considered adequate for the planned analyses. Analyses were conducted using complete cases for each model. Normality was assessed using the Shapiro–Wilk test. Because most psychometric scale and subscale scores showed significant departures from normality (*p* < 0.05), non-parametric analyses were used where appropriate [20].

Internal consistency was evaluated for each instrument using both Cronbach’s alpha and McDonald’s omega.

#### 2.3.2. Correlations and Partial Correlations

Associations among scale and subscale scores were examined using Spearman’s rank correlation coefficient. To account for potential confounding, partial correlations were additionally computed while adjusting for relevant sample characteristics (age, gender, education, work status). For the correlation matrices, *p* values were interpreted using a nominal significance threshold of α = 0.05. Given the large number of pairwise correlations, these analyses were considered exploratory and descriptive. Accordingly, isolated significant coefficients were interpreted cautiously because of the increased risk of Type I error [21].

#### 2.3.3. Regression Models

To model the association between the independent variables and symptom domains while accounting for correlations among outcomes, we fit a multivariate multiple linear regression model with the SCL-90-R subscales as a multivariate response and BIS-11, RRS, and DERS total scores entered simultaneously as independent variables [22]. This model was used to test whether each independent variable showed an overall association with the multivariate SCL-90-R symptom profile. To complement this global test and support interpretation at the level of individual symptom domains, we then estimated outcome-specific multiple linear regression models for each SCL-90-R subscale using the same set of independent variables: BIS-11 total score, RRS total score, and DERS total score. This second step allowed us to identify which specific symptom dimensions were associated with rumination, ED, and impulsiveness. Overall, multivariate significance of independent variables was evaluated using Pillai’s trace (via a multivariate test of model terms) [23]. To support interpretation at the level of individual symptom domains, we additionally estimated outcome-specific multiple linear regression models for each SCL-90-R subscale using the same set of independent variables (BIS-11 total score, RRS total score, and DERS total score). For each outcome model, we extracted unstandardized regression coefficients (B), standard errors (SE), t statistics, *p* values, and 95% confidence intervals, along with model fit indices (R^2^ and adjusted R^2^). Because multiple coefficients were tested across several symptom outcomes, *p* values from the outcome-specific regression coefficients were adjusted for multiple comparisons using the Benjamini–Hochberg false discovery rate (FDR) procedure [24]. Regression assumptions were examined using standard model diagnostics, including inspection of residual distributions, assessment of influential observations using Cook’s distance, and evaluation of multicollinearity among independent variables using variance inflation factors.

Analyses were conducted in RStudio version 4.4.3 using the packages readxl, dplyr, ppcor, Hmisc, broom, tidyr, purrr, ggplot2, and qgraph.

## 3. Results

### 3.1. Descriptive Statistics

We recruited *N* = 102 participants (mean age = 20.0 years, SD = 2.55; 51% female). Because the online survey was configured to require responses to all items, there were no missing data. All relevant sample characteristics, including descriptive statistics for scale and subscale scores, are presented in Table 1. All scales demonstrated adequate internal consistency (Appendix A, Table A1).

### 3.2. Correlations and Partial Correlations

**Figure 1:**
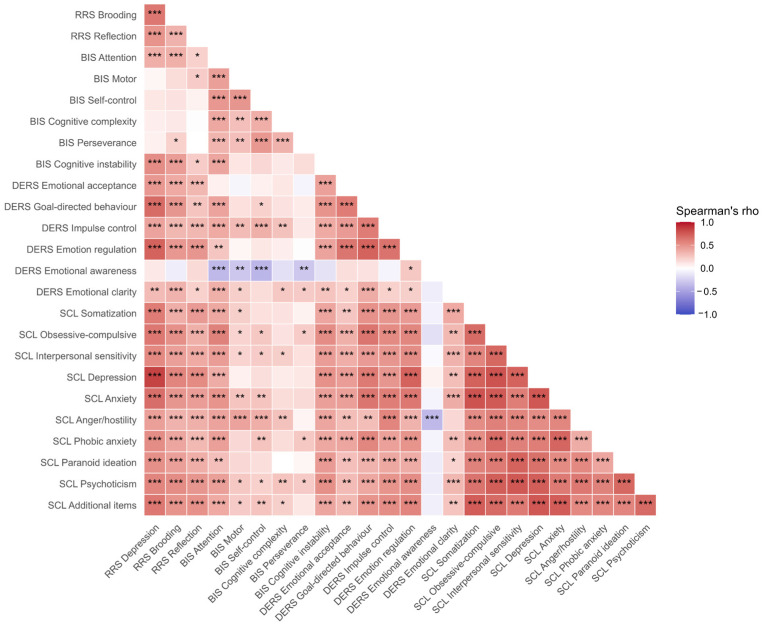
Spearman’s correlation matrix. Note: * *p* < 0.05, ** *p* < 0.01, *** *p* < 0.001. Blank cells = non-significant. *N* = 102. Color intensity reflects the direction and magnitude of Spearman’s rho, whereas asterisks indicate statistical significance. Exact rho coefficients and *p* values are provided in Supplementary Materials.

**Figure 2:**
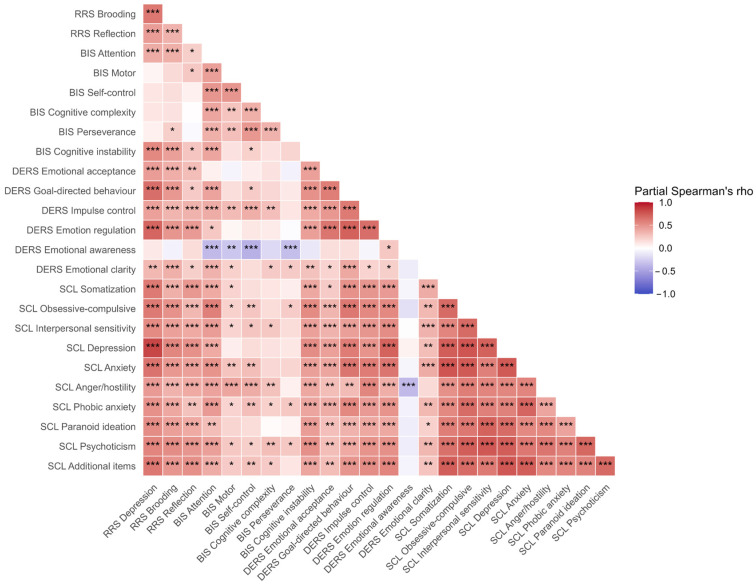
Spearman’s partial correlation matrix. Note: * *p* < 0.05, ** *p* < 0.01, *** *p* < 0.001. Blank cells represent non-significant *p*-values. Controlled for age, gender, education, and work status. *N* = 102. Color intensity reflects the direction and magnitude of Spearman’s rho, whereas asterisks indicate statistical significance. Exact rho coefficients and *p* values are provided in Supplementary Materials.

Correlations and partial correlations are presented in Figure 1 and Figure 2. The sample size for all correlation matrices was N = 102. Correlations were interpreted as follows: values of ρ ≥ 0.80 indicate a very strong relationship; ρ = 0.60–0.79, a strong relationship; ρ = 0.40–0.59, a moderate relationship; ρ = 0.20–0.39, a weak relationship; and ρ < 0.20, a very weak or negligible relationship [25]. The complete numeric correlation matrices (Spearman’s rho and corresponding *p* values) are provided as Supplementary Data (spreadsheet S1).

As shown in Figure 1, SCL-90-R symptom domains showed a consistent pattern of positive correlations with rumination and emotion-regulation difficulties. The strongest and most consistent correlations involved the RRS Depression subscale, which showed moderate-to-strong associations with SCL-90-R domains (ρ = 0.44 to 0.84, all *p* < 0.001), with the largest correlation observed for SCL-90-R Depression. Emotion-regulation difficulties showed a similar but slightly less uniform pattern, particularly for limited access to emotion-regulation strategies and goal-directed difficulties; for example, DERS Emotion Regulation was strongly correlated with SCL-90-R Depression (ρ = 0.71, *p* < 0.001), and DERS Goal-Directed Behavior Difficulties was strongly correlated with both Obsessive–Compulsive symptoms and Anxiety (ρ = 0.63, *p* < 0.001 for both). Associations with BIS-11 facets were generally less consistent and appeared more domain-specific, with the strongest associations involving Obsessive–Compulsive symptoms and BIS-11 Attention (ρ = 0.57, *p* < 0.001) and Cognitive Instability (ρ = 0.52, *p* < 0.001). A specific pattern was observed for DERS Awareness Difficulties, which showed weak-to-moderate negative correlations with selected BIS-11 domains. In the zero-order Spearman matrix, these associations were significant for BIS-11 Attention (ρ = −0.37, *p* < 0.001), Motor (ρ = −0.29, *p* = 0.003), Self-Control (ρ = −0.39, *p* < 0.001), and Perseverance (ρ = −0.29, *p* = 0.003) subscales. The same pattern was substantially confirmed in the partial correlation matrix adjusted for age, gender, education, and work status. Given their modest magnitude, these associations were interpreted cautiously and were not considered a central finding of the study. Overall, the correlation matrices were interpreted descriptively, with emphasis on the pattern and magnitude of associations rather than isolated significant coefficients.

Partial correlations produced only small changes in magnitude, and the overall pattern remained the same. The only changes in statistical significance were three previously non-significant associations that became significant after partialing: BIS-11 motor domain with SCL-90-R phobic anxiety (from *p* = 0.078 to *p* = 0.049), BIS-11 self-control subscale with BIS-11 cognitive instability (from *p* = 0.068 to *p* = 0.035), and BIS-11 cognitive complexity with SCL-90-R phobic anxiety (from *p* = 0.116 to *p* = 0.041). None of the correlations that were significant in the zero-order Spearman matrix became non-significant in the partial correlation matrix.

### 3.3. Regression Coefficients

**Figure 3:**
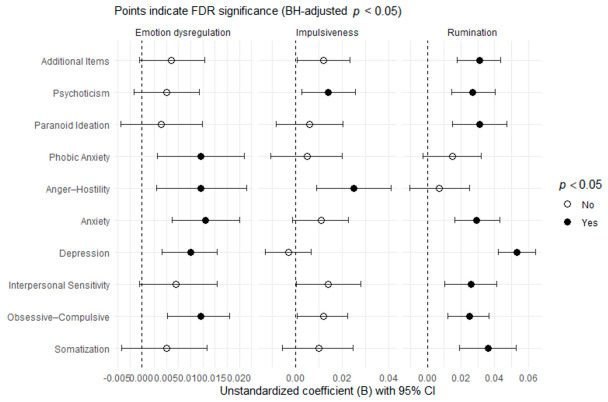
Coefficient plots from multiple regression models examining the associations of DERS, BIS-11, and RRS total scores with SCL-90-R domains. Note: points indicate statistical significance after FDR adjustment (BH-adjusted *p* < 0.05). *N* = 102.

Results of the Multivariate Multiple Regression model are reported in Figure 3.

The multivariate multiple regression indicated that all three independent variables were significantly associated with the multivariate SCL-90-R symptom profile. Pillai’s trace tests showed significant multivariate associations for BIS-11 total score, V = 0.548, F(10, 89) = 10.77, *p* < 0.001; RRS total score, V = 0.734, F(10, 89) = 24.60, *p* < 0.001; and DERS total score, V = 0.240, F(10, 89) = 2.82, *p* = 0.004. Across the outcome-specific multiple regression models, model fit ranged from adjusted R^2^ = 0.292 for Phobic Anxiety to adjusted R^2^ = 0.754 for Depression, indicating that the models explained a substantial proportion of variance in several SCL-90-R symptom domains. However, given the cross-sectional design, these coefficients should be interpreted as measures of statistical association rather than evidence of temporal or causal prediction.

After FDR correction, rumination (RRS total score) showed the broadest pattern of statistically significant associations, remaining associated with eight SCL-90-R domains: Somatization (B = 0.036, 95% CI [0.019, 0.053]), Obsessive–Compulsive (B = 0.025 [0.012, 0.037]), Interpersonal Sensitivity (B = 0.026 [0.010, 0.041]), Depression (B = 0.053 [0.042, 0.064]), Anxiety (B = 0.029 [0.016, 0.043]), Paranoid Ideation (B = 0.031 [0.015, 0.047]), Psychoticism (B = 0.027 [0.015, 0.040]), and Additional Items (B = 0.031 [0.018, 0.043]). ED (DERS total score) was also significantly (*p* < 0.05) associated with five domains: Obsessive–Compulsive (B = 0.012 [0.005, 0.018]), Depression (B = 0.010 [0.004, 0.016]), Anxiety (B = 0.013 [0.006, 0.020]), Anger–Hostility (B = 0.012 [0.003, 0.022]), and Phobic Anxiety (B = 0.012 [0.003, 0.021]). Finally, after FDR correction, impulsiveness showed more limited associations, remaining significantly (*p* < 0.05) associated only with Anger–Hostility (B = 0.025 [0.009, 0.041]) and Psychoticism (B = 0.014 [0.003, 0.026]), whereas associations with the remaining symptom domains were not statistically significant after correction. A complete table with regression coefficients is presented in Appendix A (Table A2).

## 4. Discussion

The results of our study highlight the complex interplay between ED, impulsivity, rumination, and psychopathology. The main novelty of this study is that it examined the relative contribution of three closely related but distinct transdiagnostic processes—ED, impulsiveness, and rumination—within the same clinical model in adolescents and young adults presenting for their first psychiatric outpatient consultation. The strongest associations with the depression-related rumination subscale were observed for difficulties in goal-directed behavior and limited access to effective emotion-regulation strategies, aligning with contemporary accounts of rumination as a common but maladaptive regulation strategy tied to depressive symptoms [26,27]. When people perceive limited access to effective strategies for managing negative affect, repetitive self-focused thinking may become a default response to distress, and daily life evidence likewise links emotion-regulation strategy use to rumination [28]. Similarly, distress-related disruptions in goal pursuit may be closely linked to cognitive-control difficulties, which are increasingly discussed as mechanisms connecting depressive processes to repetitive negative thinking and rumination [29]. Our results also highlight that the depression-related rumination subscale was strongly correlated with multiple SCL-90-R symptom dimensions such as somatization, obsessive–compulsive symptoms, depression, anxiety, and additional items. These findings are consistent with the view that repetitive negative thinking operates as a broad marker of psychological distress rather than a process specific to depression alone [30], and align with prior work conceptualizing rumination as a transdiagnostic factor associated with internalizing symptoms and their co-occurrence [31]. The strong association with somatization also converges with recent syntheses indicating that somatic symptom presentations are meaningfully linked to psychological processes that overlap with pathways of perseverative negative thinking [32]. Regression analyses further indicated that multiple psychopathological domains were associated with rumination, ED symptoms, and impulsiveness, with rumination showing the broadest and most consistent pattern of associations. This pattern aligns with contemporary transdiagnostic models that conceptualize repetitive negative thinking as a central mechanism cutting across internalizing symptom clusters and related complaints such as somatic distress [33]. Specifically, rumination was significantly associated with all SCL-90-R domains except phobic anxiety and anger–hostility; notably, the strongest and most consistent association for rumination was observed with depression, followed by somatization and paranoid ideation, aligning with evidence that rumination is particularly implicated in depressive symptoms, but it also might play a role in influencing physical health [34]. The associations between rumination and the SCL-90-R paranoid ideation and psychoticism dimensions should be interpreted as a secondary observation that merits replication. In the present sample, these scores reflect self-reported symptom dimensions rather than clinical diagnoses of psychotic disorders. Nevertheless, this pattern is consistent with previous evidence suggesting that repetitive negative thinking may be relevant to paranoia-like and psychotic-like experiences in young people [35]. ED symptoms showed comparatively stronger associations with phobic anxiety and anger–hostility and were also significantly associated with anxiety, depression, and obsessive–compulsive symptoms. In prior work, emotion-regulation strategies have been linked to personality traits and described as a core feature of borderline personality pathology and are also emphasized across post-traumatic stress disorders and mood disorders as a transdiagnostic vulnerability [36,37,38]. This literature suggests that ED may be especially relevant when symptoms involve high affective reactivity and difficulty returning to baseline, which fits its stronger links with threat- and anger-related complaints [39,40]. Impulsiveness showed a more domain-specific pattern, with significant associations limited to anger–hostility and psychoticism. This aligns with models that link impulsivity to broader disinhibition and externalizing liability rather than generalized internalizing distress [41]. The association with psychoticism is also plausible given evidence that impulsive behavior is elevated in psychosis-spectrum conditions and may increase clinical complexity and risk via impaired inhibitory control and stress-reactivity pathways [42,43]. Our study adopted a dimensional and transdiagnostic approach, focusing on symptom domains rather than diagnoses. Overall, these findings extend previous transdiagnostic research by showing that, in early help-seeking youth, rumination, ED, and impulsiveness do not contribute uniformly to psychopathology, but instead show partly distinct symptom-domain profiles.

### Limitations and Strengths

A key strength of this study is its clinical and developmental relevance: participants were assessed at their first outpatient psychiatric consultation in adolescence and emerging adulthood, which captures transdiagnostic processes early in help-seeking. The use of well-validated instruments with adequate reliability and an analytic approach that accounted for relevant sociodemographic covariates, while controlling multiple testing in the regression models, further strengthens confidence in the findings.

Several limitations should also be acknowledged. With regard to study design and sampling, the cross-sectional, single-site design and modest sample size constrain causal conclusions and generalizability. Moreover, because the study was based on retrospectively available clinical records, we could not reconstruct the full recruitment flow, including the total number of first-time consultations screened, excluded, or not completing the assessment; therefore, residual selection bias cannot be ruled out. In addition, the analyses were not stratified by psychiatric diagnosis, and information on comorbidities was not available, limiting the possibility of determining whether the observed associations differ across diagnostic groups or clinical profiles.

With regard to measurement, the study relied on self-report instruments, and information on current symptom severity beyond self-report measures was not available. This may have inflated associations due to shared method variance and limits the extent to which findings can be generalized to clinician-rated clinical severity. Future research should replicate these findings in larger, multi-site longitudinal studies using multimethod assessments, including clinician-rated measures, and in non-clinical settings. Such designs would help clarify the temporal ordering and potential mechanistic pathways linking rumination, ED, impulsiveness, and psychopathological symptoms. Future studies should also include measures of emotion-related impulsivity, such as urgency, which may capture affect-driven impulsive tendencies more directly than the broader BIS-11 dimensions assessed here and may show stronger associations with emotion dysregulation and psychopathological symptoms.

## 5. Conclusions

Our study is consistent with a transdiagnostic framework in which rumination, ED, and impulsiveness are associated with psychopathological symptom dimensions in adolescents and young adults presenting for first-time outpatient psychiatric consultation. Rumination showed the broadest pattern of associations, particularly with internalizing distress and related somatic complaints, whereas ED and impulsiveness showed more domain-specific associations. These findings suggest the clinical relevance of assessing self-regulatory processes early in help-seeking youth. Future longitudinal and multimethod studies are needed to test temporal ordering and clarify the mechanistic pathways linking these processes to psychopathological symptoms.

## Figures and Tables

**Table 1:** Sample characteristics and descriptive statistics for study variables.

Variable		N = 102 (%)
Gender	Female	52 (51)
	Male	41 (40.2)
	Non-binary	9 (8.8)
Education	College	3 (2.9)
	High school	62 (60.8)
	Middle school	37 (36.3)
Work status	Student	54 (52.9)
	Student and worker	8 (7.8)
	Unemployed	30 (29.4)
	Worker	10 (9.8)
	**Mean ± SD;**	**Median**
Age *	20 ± 2.55	20
BIS total score	69.67 ± 11.98	70
*Attention*	11.79 ± 3.11	12
*Cognitive complexity*	12.75 ± 2.74	12.5
*Cognitive instability* *	7.75 ± 2.14	8
*Motor*	15 ± 4.1	15
*Perseverance* *	7.25 ± 1.67	7
*Self-control* *	15.13 ± 3.64	15
DERS total score *	79.97 ± 27.49	83.5
*Emotional awareness* *	13.53 ± 5.36	14
*Emotional clarity* *	8.82 ± 2.79	9
*Emotional acceptance* *	13.01 ± 6.66	14
*Goal-directed behaviour* *	12.15 ± 3.99	13
*Impulse control* *	12.17 ± 5.1	12
*Emotion regulation* *	17.37 ± 7.63	18
RRS total score *	57.29 ± 12.72	58
*Brooding*	13.17 ± 3.03	13.5
*Depression* *	32.87 ± 8.16	33.5
*Reflection*	11.25 ± 3.44	11
SCL-90-R GSI	1.69 ± 0.76	1.74
*Anger/hostility* *	1.48 ± 1.01	1.33
*Anxiety* *	1.78 ± 0.96	1.67
*Depression* *	2.17 ± 1	2.17
*Interpersonal sensitivity*	1.79 ± 0.9	1.78
*Obsessive-compulsive*	2.04 ± 0.86	2.1
*Paranoid ideation*	1.75 ± 0.88	1.83
*Phobic anxiety* *	1.1 ± 0.92	0.7
*Psychoticism* *	1.36 ± 0.79	1.3
*Somatization* *	1.44 ± 0.97	1.33
*Additional items* *	1.68 ± 0.83	1.67

* These variables are not distributed normally (Shapiro–Wilk test < 0.05).

## Data Availability

The data presented in this study are available from the corresponding author upon reasonable request. The data are not publicly available due to privacy and ethical restrictions related to the clinical nature of the dataset and the protection of participant confidentiality.

## Supplementary Materials

The following supporting information can be downloaded at: https://www.mdpi.com/article/10.3390/brainsci16060620/s1, Table S1: Strengthening the Reporting of Observational Studies in Epidemiology (STROBE) Statement completed checklist for cross-sectional, observational studies [44]; spreadsheeet S1: The complete numeric correlation matrices (Spearman’s rho and corresponding *p* values).

## Abbreviations

The following abbreviations are used in this manuscript:

ED	Emotion dysregulation
RRS	Ruminative Response Scale
BIS-11	Barratt Impulsiveness Scale
DERS	Difficulties in Emotion Regulation Scale
SCL-90-R	Symptom Checklist-90-Revised
GSI	Global Severity Index
B	Unstandardized regression coefficients
SE	Standard error
CI	Confidence interval
FDR	False discovery rate
BH	Benjamini–Hochberg

## Appendix A

**Table A1:** Internal consistency for BIS-11, DERS, RRS, and SCL-90-R total scores.

Scale	Cronbach’s Alpha	McDonald’s Omega
BIS-11	0.82	0.82
DERS	0.86	0.88
RRS	0.91	0.91
SCL-90-R	0.97	0.97

**Table A2:** Multiple linear regression coefficients (B) and 95% C.I.s for associations of DERS, BIS-11, and RRS with SCL-90-R subscale scores.

Outcome	Independent Variable	B	SE	t	95% CI [Lower, Upper]	*p*-Value	*p*-Value (FDR Adjusted)	Adj. R^2^
Somatization	DERS	0.005	0.004	1.04	[−0.004, 0.013]	0.303	0.364	0.377
Somatization	BIS-11	0.010	0.008	1.28	[−0.005, 0.025]	0.204	0.255	0.377
Somatization	RRS	0.036	0.009	4.18	[0.019, 0.053]	<0.001	<0.001	0.377
Obsessive–Compulsive	DERS	0.012	0.003	3.60	[0.005, 0.018]	<0.001	0.002	0.584
Obsessive–Compulsive	BIS-11	0.012	0.006	2.11	[0.001, 0.023]	0.037	0.066	0.584
Obsessive–Compulsive	RRS	0.025	0.006	3.96	[0.012, 0.037]	<0.001	<0.001	0.584
Interpersonal Sensitivity	DERS	0.007	0.004	1.84	[−0.001, 0.015]	0.069	0.104	0.411
Interpersonal Sensitivity	BIS-11	0.014	0.007	2.07	[0.001, 0.028]	0.041	0.068	0.411
Interpersonal Sensitivity	RRS	0.026	0.008	3.32	[0.010, 0.041]	0.001	0.003	0.411
Depression	DERS	0.010	0.003	3.40	[0.004, 0.016]	<0.001	0.003	0.754
Depression	BIS-11	−0.003	0.005	−0.62	[−0.013, 0.007]	0.539	0.539	0.754
Depression	RRS	0.053	0.006	9.56	[0.042, 0.064]	<0.001	<0.001	0.754
Anxiety	DERS	0.013	0.004	3.74	[0.006, 0.020]	<0.001	0.001	0.603
Anxiety	BIS-11	0.011	0.006	1.81	[−0.001, 0.023]	0.073	0.104	0.603
Anxiety	RRS	0.029	0.007	4.36	[0.016, 0.043]	<0.001	<0.001	0.603
Anger–Hostility	DERS	0.012	0.005	2.61	[0.003, 0.022]	0.010	0.022	0.360
Anger–Hostility	BIS-11	0.025	0.008	3.12	[0.009, 0.041]	0.002	0.006	0.360
Anger–Hostility	RRS	0.007	0.009	0.80	[−0.011, 0.025]	0.425	0.456	0.360
Phobic Anxiety	DERS	0.012	0.005	2.68	[0.003, 0.021]	0.009	0.020	0.292
Phobic Anxiety	BIS-11	0.005	0.008	0.64	[−0.010, 0.020]	0.525	0.539	0.292
Phobic Anxiety	RRS	0.015	0.009	1.69	[−0.003, 0.032]	0.095	0.129	0.292
Paranoid Ideation	DERS	0.004	0.004	0.97	[−0.004, 0.012]	0.336	0.388	0.324
Paranoid Ideation	BIS-11	0.006	0.007	0.84	[−0.008, 0.020]	0.402	0.447	0.324
Paranoid Ideation	RRS	0.031	0.008	3.84	[0.015, 0.047]	<0.001	<0.001	0.324
Psychoticism	DERS	0.005	0.003	1.49	[−0.002, 0.012]	0.139	0.182	0.469
Psychoticism	BIS-11	0.014	0.006	2.47	[0.003, 0.026]	0.015	0.030	0.469
Psychoticism	RRS	0.027	0.006	4.24	[0.015, 0.040]	<0.001	<0.001	0.469
Additional Items	DERS	0.006	0.003	1.85	[−0.000, 0.013]	0.068	0.104	0.514
Additional Items	BIS-11	0.012	0.006	2.11	[0.001, 0.024]	0.037	0.066	0.514
Additional Items	RRS	0.031	0.006	4.73	[0.018, 0.043]	<0.001	<0.001	0.514
