# Supplementary material for: The Role of Emotion Dysregulation, Impulsiveness and Rumination in Psychopathology: Evidence from an Italian Sample of Adolescents and Young Adults
Source: Brain Sci. 2026 Jun 10;16(6):620. doi: 10.3390/brainsci16060620 (PMC13297377; doi:10.3390/brainsci16060620)
Supplement: Supplementary file 1 [file brainsci-16-00620-s001.zip › Table_S1.pdf]

Strengthening the Reporting of Observational Studies in Epidemiology (STROBE) Statement completed checklist for cross-sectional, observational studies [1]

	Item No	Recommendation	Article page
**Title and abstract**	1	(*a*) Indicate the study's design with a commonly used term in the title or the abstract	See Abstract
		(*b*) Provide in the abstract an informative and balanced summary of what was done and what was found	See Abstract
**Introduction**			
Background/rationale	2	Explain the scientific background and rationale for the investigation being reported	Section 1, p. 2
Objectives	3	State specific objectives, including any prespecified hypotheses	Section 1, p. 2-3
**Methods**			
Study design	4	Present key elements of study design early in the paper	Section 2.1, p. 3
Setting	5	Describe the setting, locations, and relevant dates, including periods of recruitment, exposure, follow-up, and data collection	Section 2.1, p. 3
Participants	6	(*a*) Give the eligibility criteria, and the sources and methods of selection of participants	Section 2.1, p. 3
Variables	7	Clearly define all outcomes, exposures, predictors, potential confounders, and effect modifiers. Give diagnostic criteria, if applicable	Section 2.1, p. 3

Data sources/ measurement	8	For each variable of interest, give sources of data and details of methods of assessment (measurement). Describe comparability of assessment methods if there is more than one group	Section 2.2, p. 3-4
Bias	9	Describe any efforts to address potential sources of bias	Section 2.1, section 2.2, p. 3
Study size	10	Explain how the study size was arrived at	Section 3.1, p. 5
Quantitative variables	11	Explain how quantitative variables were handled in the analyses. If applicable, describe which groupings were chosen and why	Section 2.3, p. 4-5
Statistical methods	12	(*a*) Describe all statistical methods, including those used to control for confounding	Section 2.3, p. 4-5
		(*b*) Describe any methods used to examine subgroups and interactions	Not applicable
		(*c*) Explain how missing data were addressed	Section 3.1, p. 5
		(*d*) If applicable, describe analytical methods taking account of sampling strategy	Not applicable
		(*e*) Describe any sensitivity analyses	Section 2.3, p. 4-5
**Results**			
Participants	13	(a) Report numbers of individuals at each stage of study—eg numbers potentially eligible, examined for eligibility, confirmed eligible, included in	Section 3.1, p. 5-6

		the study, completing follow-up, and analysed	
		(b) Give reasons for non-participation at each stage	Not applicable
		(c) Consider use of a flow diagram	Not applicable
Descriptive data	14	(a) Give characteristics of study participants (eg demographic, clinical, social) and information on exposures and potential confounders	Section 3.1, p. 5-6
		(b) Indicate number of participants with missing data for each variable of interest	Section 3.1, section 3.2, section 3.3, p. 5-8
Outcome data	15	Report numbers of outcome events or summary measures	Not applicable
Main results	16	(*a*) Give unadjusted estimates and, if applicable, confounder-adjusted estimates and their precision (eg, 95% confidence interval). Make clear which confounders were adjusted for and why they were included	Section 3.1, section 3.2, section 3.3, p. 5-8, Appendix A
		(*b*) Report category boundaries when continuous variables were categorized	Not applicable
		(*c*) If relevant, consider translating estimates of relative risk into absolute risk for a meaningful time period	Not applicable
Other analyses	17	Report other analyses done—eg analyses of subgroups and interactions, and sensitivity analyses	Appendix A
**Discussion**			

Key results	18	Summarise key results with reference to study objectives	Section 4, p. 8-9
Limitations	19	Discuss limitations of the study, taking into account sources of potential bias or imprecision. Discuss both direction and magnitude of any potential bias	Section 4, p. 8-9
Interpretation	20	Give a cautious overall interpretation of results considering objectives, limitations, multiplicity of analyses, results from similar studies, and other relevant evidence	Section 5, p. 10
Generalisability	21	Discuss the generalisability (external validity) of the study results	Section 4, p. 8-9
**Other information**			
Funding	22	Give the source of funding and the role of the funders for the present study and, if applicable, for the original study on which the present article is based	Not applicable

1. von Elm, E.; Altman, D.G.; Egger, M.; Pocock, S.J.; Gøtzsche, P.C.; Vandenbroucke, J.P.; STROBE Initiative The Strengthening the Reporting of Observational Studies in Epidemiology (STROBE) Statement: Guidelines for Reporting Observational Studies. *Lancet* **2007**, *370*, 1453–1457, doi:10.1016/S0140-6736(07)61602-X.
